# Exercise-based rehabilitation for heart failure: systematic review and meta-analysis

**DOI:** 10.1136/openhrt-2014-000163

**Published:** 2015-01-28

**Authors:** Viral A Sagar, Edward J Davies, Simon Briscoe, Andrew J S Coats, Hasnain M Dalal, Fiona Lough, Karen Rees, Sally Singh, Rod S Taylor

**Affiliations:** 1 Maidstone & Tunbridge Wells NHS Trust, Maidstone, UK; 2 South West Cardiothoracic Centre, Derriford Hospital, Plymouth, UK; 3 Peninsula Technology Assessment Group (PenTAG), University of Exeter Medical School, Exeter, UK; 4 University of East Anglia, Norwich, UK; 5 Truro & Primary Care Research Group, Department of Research and Development, Knowledge Spa, Royal Cornwall Hospitals Trust, University of Exeter Medical School, Truro, UK; 6 The Hatter Institute, UCLH NHS Trust, London, UK; 7 Division of Health Sciences, Warwick Medical School, University of Warwick, Coventry, UK; 8 Centre for Exercise and Rehabilitation Science, Glenfield Hospital, University Hospitals of Leicester NHS Trust, Leicester, UK; 9 Institute of Health Research, University of Exeter Medical School, Exeter, UK

**Keywords:** HEART FAILURE

## Abstract

**Objective:**

To update the Cochrane systematic review of exercise-based cardiac rehabilitation (CR) for heart failure.

**Methods:**

A systematic review and meta-analysis of randomised controlled trials was undertaken. MEDLINE, EMBASE and the Cochrane Library were searched up to January 2013. Trials with 6 or more months of follow-up were included if they assessed the effects of exercise interventions alone or as a component of comprehensive CR programme compared with no exercise control.

**Results:**

33 trials were included with 4740 participants predominantly with a reduced ejection fraction (<40%) and New York Heart Association class II and III. Compared with controls, while there was no difference in pooled all-cause mortality between exercise CR with follow-up to 1 year (risk ratio (RR) 0.93; 95% CI 0.69 to 1.27, p=0.67), there was a trend towards a reduction in trials with follow-up beyond 1 year (RR 0.88; 0.75 to 1.02, 0.09). Exercise CR reduced the risk of overall (RR 0.75; 0.62 to 0.92, 0.005) and heart failure-specific hospitalisation (RR 0.61; 0.46 to 0.80, 0.0004) and resulted in a clinically important improvement in the Minnesota Living with Heart Failure questionnaire (mean difference: −5.8 points, −9.2 to −2.4, 0.0007). Univariate meta-regression analysis showed that these benefits were independent of the type and dose of exercise CR, and trial duration of follow- up, quality or publication date.

**Conclusions:**

This updated Cochrane review shows that improvements in hospitalisation and health-related quality of life with exercise-based CR appear to be consistent across patients regardless of CR programme characteristics and may reduce mortality in the longer term. An individual participant data meta-analysis is needed to provide confirmatory evidence of the importance of patient subgroup and programme level characteristics (eg, exercise dose) on outcome.

## Introduction

People with heart failure (HF) experience marked reductions in their exercise capacity which has detrimental effects on their activities of daily living, health-related quality of life, and ultimately their hospital admission rate and mortality.^1^ While survival after HF diagnosis has improved, HF has a poor prognosis— 30–40% of patients diagnosed with HF die within a year.^2^ It is estimated that the total annual cost of HF to the UK National Health Service is currently around £1 billion or around 2% of the total UK health budget; approximately, 70% of this total is due to the costs of hospitalisation.^3^ ^4^ Admissions due to HF are projected to rise by 50% over the next 25 years, largely due to ageing of the population.^4^ A meta-analysis reported a mortality of 32% in HF with preserved ejection fraction (HFPEF; ejection fraction of >35–50%) versus 41% mortality in HF with reduced ejection fraction (HFREF; relative risk (RR) 0.79) over an average of 47 months follow-up.^5^ Although individuals with HFPEF contribute more than half (54%) of all patients with HF, most trials to date have recruited only patients with HFREF.^6^

Based on evidence, including the previous 2010 Cochrane review,^7^ The American College of Cardiology/American Heart Association, European Society of Cardiology, and National Institute for Health and Care Excellence (NICE) consistently recommend exercise-based cardiac rehabilitation (CR) as an effective and safe adjunct in the management of HF.^4^ ^8^ ^9^ The majority of randomised controlled trials (RCTs) included in the 2010 Cochrane review were in males at low-to-medium risk (New York Heart Association (NYHA) class II and III) and no trials including HFPEF.^7^

The aim of this updated Cochrane review was to reassess the effectiveness of exercise-based CR on mortality, hospital admissions, morbidity and health-related quality of life of patients with HF. We also sought to explore how these effects may vary across differing modes of exercise CR delivery.

## Methods

We conducted and reported this systematic review in accordance with the PRISMA (Preferred Reporting Items for Systematic Reviews and Meta-Analyses) statement.^10^

### Data sources and searches

The following databases were searched from January 2008 (the searching end date of the previous Cochrane review^10^) up to January 2013: Cochrane Central Register of Controlled Trials (CENTRAL), MEDLINE, MEDLINE In-Process, EMBASE, CINAHL and PsycINFO. Conference proceedings were searched on Web of Science. Trial registers (Controlled-trials.com and Clinicaltrials.gov) were also checked. Further trials were retrieved through a manual search of references from published included studies and recent reviews. Search filters were restricted to RCTs with no language restriction. A copy of the search strategy is available (see e-supplement).

### Study selection

Studies were eligible if they were RCTs that included adult (≥18 years) patients with HFPEF or HFREF, and reported follow-up for 6 months or more postrandomisation. Trials in which participants had previously received exercise-based CR were excluded. The intervention group should receive exercise training either alone or as a component of the comprehensive CR programme (ie, health education and/or psychological intervention in addition to exercise training). The control group must not undergo any form of exercise training but may receive active interventions (eg, education or psychological intervention) including usual medical care. Four categories of outcome were sought: (1) mortality (all-cause, HF-related and sudden death); (2) hospital admission or rehospitalisation (all-cause or HF-related); (3) health-related quality of life assessed by a validated outcome measure (eg, Short-Form 36, Minnesota Living with Heart Failure questionnaire); and (4) costs and cost-effectiveness.

Two reviewers (VAS or EJD, and RST) independently screened all titles and abstracts for eligibility. We obtained full-text copies of papers and contacted authors for more information when there was any uncertainty. Any disagreements about inclusion of studies were resolved by discussion.

### Data extraction and risk of bias assessment

The following information categories were extracted from included studies: study design, participant characteristics, intervention group details (including type frequency, duration and intensity of the exercise and cointervention received), nature of control group, length of follow-up and outcomes. We assessed trial quality using the Cochrane risk of bias tool.^11^ Study authors were contacted to seek clarification on issues of reporting, missing data or to obtain further outcome details. Data extraction and risk of bias assessment was undertaken initially by a single reviewer (VAS or EJD) using a standardised form and then verified by a second reviewer (RST), and any disagreements about interpretation of data was resolved by discussion.

### Statistical analysis

Data were analysed in accordance with the Cochrane Handbook for Systematic Reviews of Interventions.^11^ Dichotomous outcomes were expressed as RRs and 95% CIs. For continuous outcomes, we sought the mean change and SD in outcome between baseline and follow-up for both exercise and control groups, and when not available, the absolute mean (and SD) outcome at follow-up for both groups was used. We calculated mean difference (MD) or standardised MD (SMD), and 95% CI for each study. Heterogeneity between studies was assessed qualitatively (by comparing the characteristics of included studies) and quantitatively (using the I^2^ statistic). The results from included studies were combined for each outcome to give an overall estimate of the treatment effect where appropriate. A fixed-effect model meta-analysis was used except where statistical heterogeneity was identified (I^2^ statistic >50%) and a random-effect model used.

We explored the potential heterogeneity in the effect of exercise-based CR by two approaches: (1) within trial: subgroup analyses (supported by subgroup×treatment group interaction term), and (2) between trial: metaregression. Metaregression was used to examine the association between the effect of exercise on all-cause mortality, all-cause hospitalisation and health-related quality of life up to 12 months (as these three outcomes contained the most trials). Specific study level covariates included in meta-regression analyses included: dose of aerobic exercise (calculated as the overall number of weeks of training multiplied by the average number of sessions per week multiplied by the average duration of sessions in minutes); type of exercise (aerobic training alone or aerobic plus resistance training); setting (hospital only, home only, or both hospital and home); type of rehabilitation (exercise only or comprehensive); overall risk of bias (‘low’, ie, absence of bias in ≥5 of 8 of the risk of bias items or ‘high’—absence of bias in <5 of 8 of the risk of bias items); single or multicentre; and publication date. Given the relatively small ratio of trials to covariates, metaregression was limited to univariate analysis and run using the STATA permute option.^11^ Funnel plots and Egger tests were used to assess potential small study effects and publication bias for those outcomes with an adequate number of trials.^12^ All statistical analyses were performed using RevMan V.5.2 and STATA V.13.0.

## Results

### Identification and selection of studies

**Figure 1:**
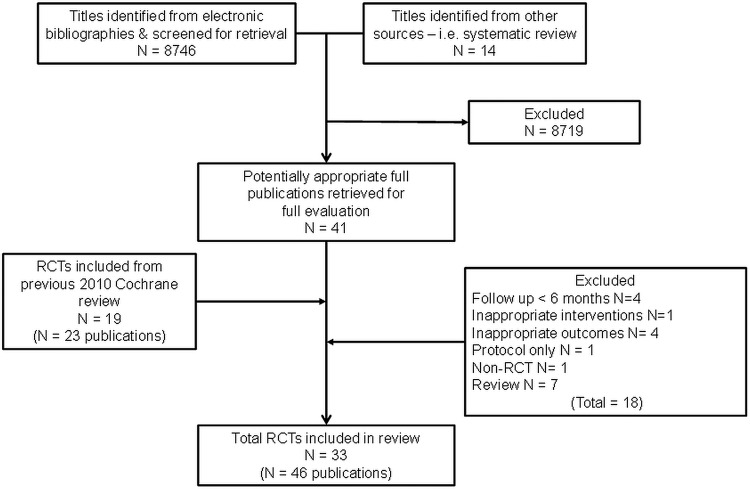
Summary of study inclusion/exclusion process.

Our searches yielded 8746 titles. Following the review of titles and abstracts, we included 41 full papers. Eighteen of these papers were excluded resulting in 14 RCTs (23 papers) meeting the review inclusion criteria. The previous 2010 Cochrane review 2010 provided 19 RCTs (23 papers) giving an overall total of 33 included RCTs (46 papers; citations available, see e-supplementary II^13–58^). Figure 1 summarises the study selection process. Two trials were each split into two subcomparisons as both randomised patients to two different exercise interventions.^26^ ^44^

### Description of included trials

The 33 trials randomised a total of 4740 patients predominantly with HFREF and NYHA class II and III (see table 1). Detailed study characteristics are available (see e-supplement). Four more recent trials included a (undefined) number of patients with HFPEF.^22^ ^26^ ^49^ ^54^ The majority of trials were small (26 trials <100 participants) and single centre (30 trials), with 1 large trial (HF-ACTION 2009) contributing approximately 50% (2331 participants) of all included patients.^33–39^ The mean age of patients across the trials ranged from 51 to 81 years. Although there was evidence of more females recruited in recent trials, the majority of patients were predominantly male (median 87%). Eleven trials reported follow-up in excess of 12 months.^13^ ^16^ ^18^ ^22^ ^35^ ^46–49^ ^54^ Details of intervention and control were often poorly reported. All trials evaluated an aerobic intervention and 11 also included resistance training.^13^ ^20^ ^23^ ^40^ ^41^ ^45^ ^46^ ^50^ ^56^ ^57^ Exercise training was most commonly delivered in either an exclusively centre-based setting or a centre-based setting in combination with some home exercise sessions. Five studies were conducted in an exclusively home-based setting.^23^ ^26^ ^40^ ^52^ ^54^ The dose of exercise training ranged widely across studies with session duration of 15–120 min, 1–7 sessions/week, intensity of 40–80% of maximal heart rate (or equivalent of 50–85% of maximal oxygen uptake (VO_2_ max) or Borg rating of 12–18), and delivered over a duration of 15–120 weeks.

**Table 1:** Selected characteristics of the 33 included trials

Characteristic	Number (%) or median (range)
Exercise-only CR	10 (30)
Setting	
Centre-based	14 (43)
Home-based	5 (15)
Both	13 (39)
Unspecified	1 (3)
Sample size	52 (19–2331)
Publication date	
1990–1999	5 (15)
2000–2009	22 (66)
2010 or later	6 (18)
Single centre	30 (91)
Study location	
Europe	20 (60)
North America*	11 (33)
Other	2 (6)
Sex	
Men only	12 (36)
Women only	0 (0)
Both	20 (61)
Unspecified	1 (3)
Age (years)	60.5 (51–81)
Diagnosis	
HFREF only	29 (88)
HFPEF only	0 (0)
Both	4 (12)
Left ventricular ejection fraction (%)	29 (21–41)
Included NYHA IV	6 (18)
Unspecified	4 (12)

*HF-ACTION trial also included six French centres (out of 82 centres): +median of study means.

CR, cardiac rehabilitation; HFPEF, heart failure with preserved ejection fraction; HFREF, heart failure with reduced ejection fraction; NYHA, New York Heart Association.

### Risk of bias

A number of trials (particularly those published prior to 2000) failed to give sufficient details to allow complete assessment of their potential risk of bias (table 2). However, where details were reported, the overall risk of bias of included studies was judged as moderate. Only 6 trials provided an adequate description of the randomisation process.^13^ ^20^ ^35^ ^40^ ^46^ ^58^ Nevertheless, none of the studies had imbalance in baseline characteristics. Although not always explicitly stated, most studies appeared to perform intention-to-treat analysis, comparing exercise and control group according to initial random allocation. Given the nature of interventions, it was not possible to blind the participants and care-givers, though a number of studies did report blinding of outcome assessment.^22^ ^26^ ^35^ ^45^ ^46^ ^49^ ^55^ ^58^ We found no evidence of selective outcome reporting. Some studies may be prone to performance bias as they failed to report cointervention details for both exercise and control groups.^16^ ^27^ ^28^ ^30^ ^42^ ^43^ ^46^ ^49^ ^53^ Only two studies failed to report losses to follow-up or drop-out rates.^19^ ^55^

**Table 2:** Risk of bias assessment of included studies

Author (year)	Adequate sequence generation	Allocation concealment	Outcome blinding	Intention-to- treat analysis	Groups balanced at baseline	Complete outcome reported
Austin (2005)	√	√	X	√	√	√
Belardinelli (1999)	?	?	?	?	√	√
Belardinelli (2012)	?	?	?	√	√	√
Bocalini (2008)	?	?	?	X	√	√
DANREHAB (2008)	√	√	√	√	√	√
Davidson (2010)	√	?	√	√	√	√
Dracup (2007)	?	?	?	√	√	√
Gary (2010)	?	?	√	√	√	√
Giannuzzi (2003)	?	?	?	√	√	√
Gielen (2003)	?	?	?	√	√	√
Gottleib (1999)	?	?	?	?	√	√
Hambrecht (1995)	?	?	?	?	√	√
Hambrecht (1998)	?	?	?	√	√	√
Hambrecht (2000)	√	?	?	√	√	√
HF-ACTION (2009)	√	√	√	√	√	√
Jolly (2009)	√	√	X	√	?	√
Jónsdóttir (2006)	?	?	?	√	√	√
Keteyian (1996)	?	?	?	√	√	√
Kletcha (2007)	?	?	?	?	√	√
Klocek (2005)	?	?	?	?	√	√
Koukouvou (2004)	?	?	√	?	√	√
McKelvie (2002)	√	√	√	?	√	√
Mueller (2007)	?	?	?	?	√	√
Myers (2000)	?	?	?	√	√	√
Nilsson (2008)	?	?	√	√	√	√
Normal (2012)	?	?	X	√	√	√
Passino (2006)	?	?	?	?	√	√
Pozehl (2007)	?	?	?	?	√	√
Wall (2010)	?	?	?	√	√	√
Willenheimer (2001)	?	?	√	?	√	√
Witham (2005)	√	?	√	√	√	√
Witham (2012)	√	√	?	√	√	√
Yeh (2011)	√	?	√	√	√	√

√, risk of bias criteria met; X, risk of bias criteria not met; ?, inadequate reporting to assess risk of bias criteria.

HF, heart failure.

### Outcomes

#### Mortality

**Figure 2:**
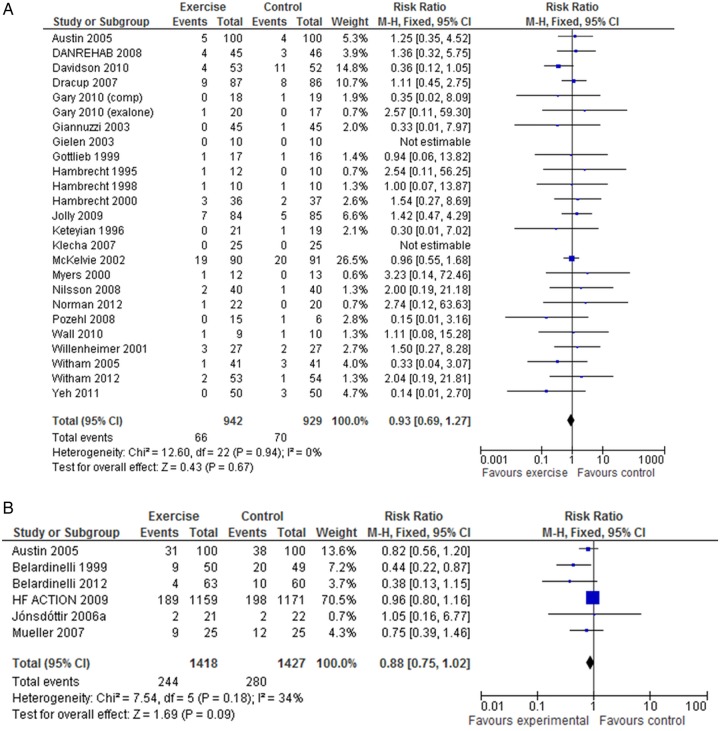
(A) Pooled all cause mortality for trials up to 12 months follow up. (B) Pooled all cause mortality for trials with more than 12 months follow up.

There was no significant difference in pooled mortality up to 12 months follow-up between exercise training and control groups (25 trials, fixed-effect RR 0.92, 95% CI 0.67 to 1.26, p=0.67; I^2^=0%, figure 2A). There was a trend towards a reduction in all-cause mortality when pooled across longest follow-up point of the six trials with more than 12 months follow-up (fixed-effect RR 0.80, 95% CI 0.75 to 1.02; p=0.09; I^2^=34%, figure 2B). Few studies consistently reported deaths due to HF or sudden death.

#### Hospital admissions

**Figure 3:**
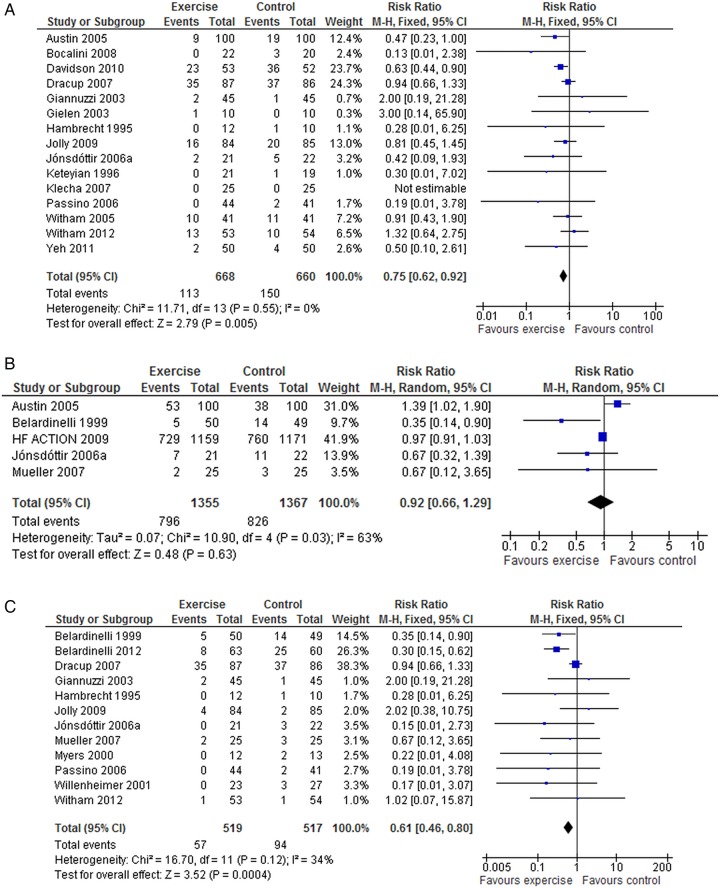
(A) Pooled all hospitalisations up to 12 months follow up. (B) Pooled all hospitalisations more than 12 months follow up. (C) Pooled heart failure hospitalisations.

There were reductions in the number of patients experiencing all hospital admissions with exercise compared with control up to 12 months follow-up (15 trials, fixed-effect RR 0.75, 95% CI 0.62 to 0.92; p=0.005; I^2^=0%, figure 3A) and HF-specific admissions (12 trials, fixed-effect RR 0.61, 95% CI 0.46 to 0.8, p=0.0004; I^2^=34%, figure 3C).There was no difference in all hospital admissions in trials with more than 12 months follow-up (5 trials, random-effect RR 0.92, 95% CI 0.66 to 1.29, p=0.63; I^2^=63%, figure 3B).

#### Health-related quality of life

**Figure 4:**
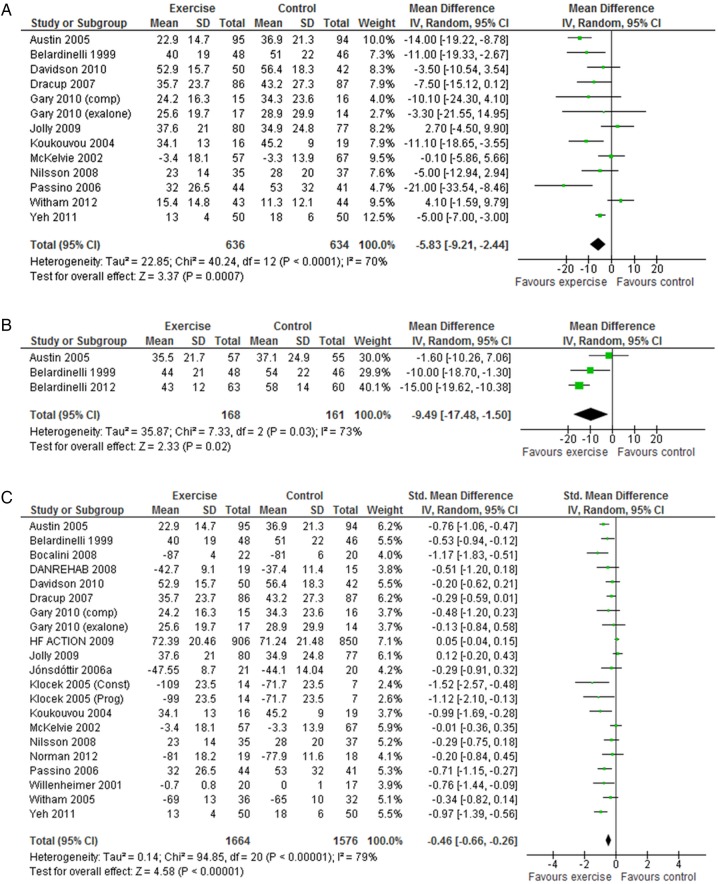
(A) Pooled Minnesota Living with Heart Failure score up to 12 months follow up. (B) Pooled Minnesota Living with Heart Failure score more than 12 months follow up (C) All quality of life scores up to 12 months follow up.

A total of 18 trials reported a validated health-related quality of life measure. While majority of studies (13 studies) used the disease-specific Minnesota Living with Heart Failure questionnaire, the HF-ACTION trial used Kansas City Cardiomyopathy Questionnaire.^33^ Generic health-related quality of life was assessed using the EuroQoL, Short-Form 36, Psychological General Wellbeing index, Patient’s Global Assessment of Quality of life and Spritzer’s Quality of Life Index. Eleven of 18 trials (61%) reported superior health-related quality of life at follow-up in the exerciser group compared with controls and in no case was health-related quality of life score lower with exercise than control (table 3). Across the studies reporting the total Minnesota Living with Heart Failure questionnaire score up to 12 months follow-up, there was evidence of a clinically important improvement with exercise (random-effect MD −5.8, 95% CI −9.2 to −2.4; p=0.0007; I^2^=70%, figure 4A). This benefit was also seen in the three trials that reported follow-up of more than 12 months (random-effect MD −9.5, 95% CI −17.5 to −1.5; p=0.022; I^2^=73%, figure 4B). Pooling across all studies, regardless of outcome measure used, showed a significant improvement in quality of life with exercise (random-effect SMD −0.46, 95% CI −0.66 to −0.26; p<0.0001; I^2^=79%, figure 4C). Where studies reported more than one health-related quality of life measure score, we randomly selected a single score for meta-analysis to prevent double counting of a study. The inference of our analysis did not change when we selected an alternative health-related quality of life score.

**Table 3:** Health-related quality of life results

Trial first author (year)	Follow-up	Measure	Outcome values (or change from baseline) at follow-up Mean (SD) Control versus exercise, between group p value	Between-group difference
Austin (2005)	6 months	MLWHF		
		Physical	20.4 (12.2) vs 12.6 (9.7) p<0.0001*	Exercise>control
		Emotional	8.0 (7.1) vs 4.4 (10.4) p<0.01*	Exercise>control
		Total	36.9 (24.0) vs 22.9 (17.8) p<0.001*	Exercise>control
		EQ-5D	0.58 (0.19) vs 0.70 (0.16) p<0.0001*	Exercise>control
	5 years	MLWHF		
		Physical	19.3 (23.5) vs 18.3 (11.2) p=0.66*	Exercise=control
		Emotional	7.6 (7.1) vs 7.4 (6.5) p=0.88*	Exercise=control
		Total	37.1 (24.9) vs 35.5 (21.7) p=0.72*	Exercise=control
		EQ-5D	0.58 (0.22) vs 0.64 (0.19) p=0.12*	Exercise=control
Bellardinelli (1999)	15 months	MLWHF total	52 (20) vs 39 (20) p<0.001	Exercise>control
	29 months		54 (22) vs 44 (21) p<0.001	Exercise>control
DANREHAB (2008)	12 months	SF-36		
		PCS	37.4 (11.4) vs 42.7 (9.1)* p=0.14	Exercise=control
		MCS	50.5 (10.0) vs 49.7 (8.8)* p=0.81	Exercise=control
Davidson (2010)	12 months	MLWHF total	56.4 (18.3) vs 52.9 (15.7) p=0.33	Exercise=control
Dracup (2007)	6 months	MLWHF		
		Physical	19.4 (11.5) vs 16.1 (10.0) p=0.04*	Exercise>control
		Emotional	10.5 (7.4) vs 7.8 (6.6) p=0.01*	Exercise>control
		Total	43.2 (26.5) vs 35.7 (23.7) p=0.05	Exercise>control
Gary (2010) Comp	6 months	MLWHF total	34.3 (23.6) vs 24.2 (16.3) p=0.18*	Exercise=control
Gary (2010) Exer	6 months	MLWHF total	28.9 (29.9) vs 25.6 (19.7) p=0.71*	Exercise=control
Gottlieb (1999)	6 months	MLWHF		
		Total	NR (NR) vs 22 (20) NR	NR
		MOS		
		PF	NR (NR) vs 68 (28) NR	NR
		RL	NR (NR) vs 50 (42) NR	NR
		GH	NR (NR) vs 361 (224) NR	NR
HF-ACTION (2009)	3 months	KCCQ+	5.21 (95% CI 4.42 to 6.00) vs 3.28 (2.48 to 4.09) p<0.001	Exercise>control
Jolly (2009)	6 months	MLWHF total	34.5 (24.0) vs 36.3 (24.1) p=0.30	Exercise=control
		EQ-5D	0.62 (0.32) vs 0.66 (0.24) p=0.004	Exercise>control
		MLWHF total	34.9 (24.8) vs 37.6 (21.0) p=0.80	Exercise=control
	12 months	EQ-5D	0.69 (0.28) vs 0.68 (0.21) p=0.07	Exercise=control
Jónsdóttir (2006)	6 months	Icelandic quality of life questionnaire	4.10 (14.04) vs 47.55 (8.7) p=0.34	Exercise=control
Klocek (2005)	6.5 months	PGWB total	99.0 vs 109.0 (training grp constant) vs 71.7 (training grp progressive) p<0.01	Exercise>control
Koukouvou (2004)	6 months	MLWHF total	34.1 (13.0) vs 45.1 (9.9) p=0.05*	Exercise>control
		Spritzer QLI total	7.1 (1.1) vs 9.1 (1.1) p<0.0001*	Exercise>control
McKelvie (2002)	12 months	MLWHF total+	−3.3 (13.9) vs −3.4 (18.1) p=0.98	Exercise=control
Nilsson (2008)	12 months	MLWHF total	28 (20) vs 22 (12) p=0.003	Exercise>control
Norman (2012)	6 months	KCCQ	77.9 (11.6) vs 81.0 (18.2) p=0.78	Exercise=control
Passino (2006)	9.75 months	MLWHF total	53 (32) vs 32 (26.5) p<0.0001*	Exercise>control
Willenheimer (2001)	10 months	PGAQoL	0 (1) 0.7 vs (0.9) p=0.023	Exercise>control
Witham (2005)	6 months	GCHFQ	69 (13) vs 65 (10) p=0.48	Exercise=control
Yeh (2011)	12 months	MLWHF total	18 (6) vs 13 (4) p<0.0001	Exercise>control

Exercise=control: no statistically significant difference (p>0.05) in HRQoL between exercise and control groups at follow-up.

Exercise>control: statistically significant (p≤0.05) higher HRQoL in exercise compared with control group at follow-up.

Exercise<control: statistically significant (p≤0.05) lower HRQoL in exercise versus control group at follow-up.

*p Values calculated by authors of this paper;+: change in outcome from baseline.

comp, comprehensive cardiac rehabilitattion; exer, exercise only cardiac rehabilitation; EQ-5D, EuroQoL; GCHFQ, Guyatt Chronic Heart Failure Questionnaire; GF, general health; HRQoL, health-related quality of life; KCCQ, Kansas City Cardiomyopathy Questionnaire; MCS, Mental Component Score; MLWHF, Minnesota Living with Heart Failure questionnaire; NR, not reported; PCS, Physical Component Score; PF, physical functioning; PGAQoL, Patient’s Global Assessment of Quality of Life; PGWB, Psychological General Wellbeing Index; QLI, quality of life index; RL: role limitation; SF-36, Short-Form 36.

#### Cost and cost-effectiveness

Three studies reported economic data, two undertaking a formal cost effectiveness analysis^17^ ^37^ and one reported costs^57^ (see e-supplementary table). Based on the Belardinelli trial, Georgiou *et al*^17^ estimated an additional mean healthcare cost in the training group compared with controls of US$3227/patient. Using exponential survival modelling to 15.5 years, the estimated increment in life expectancy with exercise was 1.82 years/person compared with patients in the control group and an incremental cost-effectiveness ratio of US$1773/life-year saved. The HF-ACTION group estimated a small mean gain in quality-adjusted life-years of 0.03 at an additional mean cost of US$1161/patient at 2.5 years follow-up.^37^ Witham *et al*^57^ reported the mean cost in the exercise group were lower (£477.85/patient) than the control group at 6 months follow-up. This cost difference was primarily the result of a reduction in the days of hospital admission in the exercise group compared with controls. None of the between group differences in costs or outcomes across these three studies achieved statistical significance at the p≤0.05 level.

### Exploration of heterogeneity

There were no significant associations in univariate metaregression for all-cause mortality, all hospitalisation and health-related quality of life with the exception of risk of bias and the setting for health-related quality of life (table 4). The mean effect size for studies with a higher risk of bias was larger than that for studies with lower risk of bias (Minnesota Living with Heart Failure MD: high risk −14.4 vs low risk −4.2, p=0.04): and higher for single centre studies (all health-related quality of life measures SMD: single centre −0.90 vs multicentre −0.35, p=0.04).

**Table 4:** Univariate metaregression results

	All-cause mortality p Value	All hospitalisations p Value	MLWHF p Value	All HRQoL outcomes p Value
Type of rehabilitation (exercise only vs comprehensive)	0.76	0.77	0.23	0.28
Type of exercise (aerobic training alone vs aerobic plus resistance training)	0.74	0.56	0.28	0.54
Exercise dose (number of weeks×number of sessions/week×average duration of session in hours)	0.15	0.80	0.15	0.28
Exercise setting (hospital only, home only, both hospital and home)	0.23	0.11	0.85	0.23
Single versus multicentre	0.94	0.70	0.14	0.01
Publication date	0.54	0.54	0.46	0.60
Risk of bias*	0.40	0.57	0.04	0.08

*‘Low’ risk of bias trial: absence of bias in >5 out 8 of risk of bias items vs ‘high’ risk of trial: absence of bias in <5 out 8 items.

HRQoL, health-related quality of life; MLWHF, Minnesota Living with Heart Failure questionnaire.

Although, a number of studies reported that they had undertaken subgroup analyses, the methods used were often unclear (see e-supplementary table). Only the large HF-ACTION trial stated they performed a predefined interaction test of differences in intervention effects between subgroups. The HF-ACTION authors reported no evidence of difference in the intervention effects as assessed on either the primary outcome (all-cause mortality or hospitalisation) or health-related quality of life (Kansas City Cardiomyopathy Questionnaire overall score) across a number of patient-defined subgroups.^34^ ^35^ ^37^ A post hoc analysis by the HF-ACTION group showed that a minimum volume of exercise (3–7 metabolic equivalent-hours per week) needed to be undertaken by patients for them to achieve a clinical benefit.^34^

### Small study bias

There was no evidence of funnel plot asymmetry for all-cause mortality (Egger test p=0.805, e-supplementary figure) or Minnesota Living with Heart Failure (Egger test p=0.606, e-supplementary figure). The funnel plots for SMD health-related quality of life showed evidence of asymmetry (Egger test p<0.0001, e-supplementary figure).

## Discussion

This systematic review shows that when compared to the no-exercise control, exercise interventions alone or as a component of comprehensive CR programme does not reduce or increase short-term (up to 12 months follow-up) all-cause mortality. We saw reductions in the risk of hospitalisation due to HF (RR reduction: 25%, 95% CI 8% to 38%) and improvements in health-related quality of life following exercise interventions. In trials reporting the Minnesota Living with Heart Failure questionnaire, those undertaking exercise were on an average 5.8 points higher than controls. A difference of four points or larger on the Minnesota Living with Heart Failure questionnaire has been shown to represent a clinically important, meaningful difference for patients.^59^ While the majority of included participants in this review were HFREF and NYHA class II and III, recent trials have recruited those who with HFPEF and NHYA IV, and a greater proportion of females and older patients. We found the benefits of exercise-CR appear to be independent of type of exercise CR (exercise only vs comprehensive CR, aerobic exercise only vs aerobic and resistance exercise, average dose of exercise intervention), and trial characteristics (ie, length of follow-up, overall risk of bias, publication date).

Many trials included in this review have been conducted in the era of contemporary medical therapy for HF. For example, in the large multicentre HF-ACTION trial, 94% of patients were receiving β-blockers and angiotensin-receptor blocker or ACE inhibitors, and 45% had an implantable cardioverter defibrillator or implanted biventricular pacemaker at the time of enrolment.^35^ Given the proven survival advantage of these medical treatments, it might be expected that any incremental all-cause mortality benefit with exercise is likely to be small. Nevertheless, there was a trend (p=0.09) towards a reduction in all-cause mortality with exercise training in the six trials reporting outcomes beyond 12 months.

The improvements in health-related quality of life with exercise training seen in this review are in accordance with the previous systematic review of van Tol *et al*^60^ but not with that of Chien *et al*,^61^ which focused on home-based exercise training and concluded that exercise training compared with usual care did not improve the health-related quality of life of patients with HF. However, the review by Chien *et al* was limited to three trials in 198 patients. Our metaregression analysis showed no difference in the magnitude of the reduction in hospitalisations and improvement in health-related quality of life with exercise training in those studies based in a hospital setting compared with those based in a home setting.

### Study limitations

The general lack of reporting of methods in the included RCT reports made it difficult to assess their methodological quality and thereby, judge their risk of bias. There appeared to be improvement in the quality of reporting in recent trials. Funnel plot asymmetry for health-related quality of life outcomes indicated evidence of small study bias and therefore, possible publication bias. Future trials need to also provide fuller details of the interventions and controls in accordance with Consolidated Standards of Reporting Trials (CONSORT) extension for trials assessing non-pharmacological treatments.^62^ A specific goal of this update review was to clarify the impact of exercise training programmes on clinical events; many included trials were relatively small and of short-term follow-up so that the number of deaths and hospitalisations reported by the majority of trials was small. The majority of trials reported low numbers of deaths and hospitalisations. There was evidence of larger treatment effect for health-related quality of life outcomes in studies judged to be at higher risk of bias compared with lower risk of bias studies, suggesting that risk of bias may be a major driver of the substantive statistical heterogeneity seen across trials in this outcome. The majority of trials in this review have investigated exercise training as a single intervention and against a no-exercise control. However, in practice, exercise-based CR is often an adjunct to other HF management interventions, such as specialist HF nurse support or disease management programmes. While trials have demonstrated the benefits of such HF management interventions alone, few trials have compared such interventions with and without adding a structured exercise training programme.^63^ ^19^ This is an important question for the future design of HF services because the addition of an exercise CR programme can add considerably to staffing and equipment costs. An individual participant data meta-analysis (ExTraMATCH II), using the RCTs identified in this review, is currently underway to clarify the patient and intervention characteristics that may drive variation in outcomes with exercise-based CR for HF.^64^

In conclusion, this updated Cochrane review shows that improvements in hospitalisation and health-related quality of life with exercise-based CR appear to be consistent across both patients with HF regardless of type of CR programme and may reduce mortality in the longer term. Individual participant data meta-analysis is needed to provide confirmatory evidence of the importance of patient subgroup and exercise programme characteristics on outcomes.

In addition to improving the quality of reporting, future clinical trials of exercise-based interventions in HF need to consider the generalisability of trial populations (women, older people and people with HFPEF remain under-represented in trial populations) and interventions to enhance the long-term maintenance of exercise-based CR programmes, as well as the outcomes, costs and cost-effectiveness of programmes delivered exclusively in a home-based setting.
